# Biodegradable polylactic acid microplastics rewire the soil food web by impeding amoebae predation

**DOI:** 10.1093/ismejo/wrag237

**Published:** 2026-09-12

**Authors:** Lin Zhang, Fei Liu, Lu Ma, Min Zhou, Yuanchen Zhao, Yiyan Yin, Bo Wu, Longfei Shu

**Affiliations:** School of Environmental Science and Engineering, Southern Marine Science and Engineering Guangdong Laboratory (Zhuhai), Guangdong Provincial Key Laboratory of Environmental Pollution Control and Remediation Technology, State Key Laboratory for Biocontrol, Sun Yat-sen University, Guangzhou 510006, China; School of Environmental Science and Engineering, Southern Marine Science and Engineering Guangdong Laboratory (Zhuhai), Guangdong Provincial Key Laboratory of Environmental Pollution Control and Remediation Technology, State Key Laboratory for Biocontrol, Sun Yat-sen University, Guangzhou 510006, China; School of Environmental Science and Engineering, Southern Marine Science and Engineering Guangdong Laboratory (Zhuhai), Guangdong Provincial Key Laboratory of Environmental Pollution Control and Remediation Technology, State Key Laboratory for Biocontrol, Sun Yat-sen University, Guangzhou 510006, China; School of Environmental Science and Engineering, Southern Marine Science and Engineering Guangdong Laboratory (Zhuhai), Guangdong Provincial Key Laboratory of Environmental Pollution Control and Remediation Technology, State Key Laboratory for Biocontrol, Sun Yat-sen University, Guangzhou 510006, China; School of Environmental Science and Engineering, Southern Marine Science and Engineering Guangdong Laboratory (Zhuhai), Guangdong Provincial Key Laboratory of Environmental Pollution Control and Remediation Technology, State Key Laboratory for Biocontrol, Sun Yat-sen University, Guangzhou 510006, China; School of Environmental Science and Engineering, Southern Marine Science and Engineering Guangdong Laboratory (Zhuhai), Guangdong Provincial Key Laboratory of Environmental Pollution Control and Remediation Technology, State Key Laboratory for Biocontrol, Sun Yat-sen University, Guangzhou 510006, China; School of Environmental Science and Engineering, Southern Marine Science and Engineering Guangdong Laboratory (Zhuhai), Guangdong Provincial Key Laboratory of Environmental Pollution Control and Remediation Technology, State Key Laboratory for Biocontrol, Sun Yat-sen University, Guangzhou 510006, China; School of Environmental Science and Engineering, Southern Marine Science and Engineering Guangdong Laboratory (Zhuhai), Guangdong Provincial Key Laboratory of Environmental Pollution Control and Remediation Technology, State Key Laboratory for Biocontrol, Sun Yat-sen University, Guangzhou 510006, China

**Keywords:** amoebae, polylactic acid (PLA), biodegradable plastics (BDPs), microplastics, *Dictyostelium discoideum*

## Abstract

Soil amoebae function as both keystone microbial predators and sensitive indicators of soil ecosystem health. Although polylactic acid (PLA) biodegradable plastics are increasingly adopted to reduce persistent plastic pollution, their ecological impacts, particularly during the active degradation phase, remain poorly understood. In this study, we established multiple co-culture interfaces to investigate the interactions and effects between soil amoebae and polylactic acid microplastics (PLA MPs). Our results show that PLA MPs fundamentally reshape amoeboid behavioral strategies and restructure soil microbial networks. Specifically, PLA MPs induce particle-size-dependent shifts in microbial community composition and functional potential, trigger strong avoidance behavior in amoebae that impairs its chemotactic capacity to locate bacterial prey, and disrupt the development of the PLA-associated plastisphere by suppressing microbial interactions. PLA MPs also impose significant fitness costs on amoebae, reducing spore production and altering developmental morphology. This study establishes soil amoebae as powerful bioindicators for evaluating the ecological impact of biodegradable plastics and demonstrates that PLA-driven disruption arises not only from its persistence but also from its degradation intermediates. These findings uncover hidden ecological trade-offs in the adoption of biodegradable plastics and offer key mechanistic insights to support environmental risk assessment.

## Introduction

Following the pervasive integration of microplastics into marine and terrestrial food webs [1–3], research efforts have intensified to elucidate the ecological impacts of escalating particulate concentrations across trophic levels [4]. Conventional petroleum-derived plastics, including polyethylene (PE), polypropylene (PP), polystyrene (PS), and polyvinyl chloride (PVC), are the primary sources of microplastics [5]. Microplastics elicit multidirectional interactions with terrestrial microbiomes, reconfiguring microbial community structure and metabolic potentials [6]. They also perturb soil physiochemistry and nitrogen cycling [7]. To mitigate non-degradable microplastic pollution, biodegradable plastics (BDPs) have been developed as promising alternatives [8]. In 2025, the global production capacity of biobased plastics reached 2.31 million tonnes, with biobased and BDPs accounting for 46.8% of this capacity, or ~1.08 million tonnes (European Bioplastics, https://www.european-bioplastics.org/market, accessed on 27 January 2026). Common types of BDPs include polylactic acid (PLA), poly (butylene adipate-co-terephthalate) (PBAT), polyhydroxyalkanoates, polycaprolactone, and polybutylene succinate, which exhibit distinct material properties and environmental behaviors [9].

PLA represents the largest share within the biodegradable segment, at 26.4%. Because PLA is a pioneering BDP derived from renewable resources like corn starch [10], it is recognized for its potential to enzymatically degrade into CO₂ and biomass under ideal conditions [11]. This makes BDP mulch the primary application form of PLA in agriculture, widely deployed in various scenarios ranging from controlled environments such as greenhouses to traditional open-field farms [12, 13]. The widespread use of PLA offloads significant ecological risks onto soil systems. Studies have confirmed that PLA and its blends with PBAT can disrupt soil carbon cycling by stimulating CO_2_ efflux whereas concurrently destabilizing microbial community structures [14, 15]. Although PLA disrupts soil microbiota and transmits effects from below- to above-ground [16, 17], current research focuses predominantly on prokaryotes such as bacteria, neglecting key microbial predators governing soil ecological processes [18]. This creates a critical gap in identifying relevant microbial benchmarks to assess their biological impact. Such an evaluation must extend beyond bacteria to include keystone microbial predators, particularly soil protists like amoebae, that govern soil ecological processes.

Among all soil protists, amoebae stand out as a typical and unique group. By driving coordinated shifts in microbial community structure and function, amoebae emerge as key regulators of nutrient mineralization, particularly for nitrogen and phosphorus [19–21]. Through the microbial loop, they feed on bacteria and convert bacterial nitrogen into plant-available ammonium released near roots, thereby replenishing the soil ammonium (NH_4_^+^) pool [22, 23]. Ammonium is a major nitrogen source for plants, with 42% of terrestrial plant nitrogen derived from this pool globally [24], indicating that amoebae predation is directly linked to soil functioning and energy flow. In natural soils, amoebae preferentially prey on high-nutrition bacteria, especially Gram-negative and non-motile strains [25]. This predation suppresses pathogens like *Ralstonia solanacearum* and enriches beneficial bacteria such as *Bacillus velezensis* [26]. Certain rhizosphere bacteria even colonized amoebae as endosymbionts, forming synergistic functional units [27]. In this context, persistent PLA fragments act as continuously evolving interfaces that host and shape microbial responses, exerting long-term negative effects [28]. Compounding this concern, amoebae predation heavily depends on chemotaxis mediated by chemical signals [29], yet the incomplete mineralization of PLA in natural soils produces oligomers that may interfere with this chemotactic process [13, 16]. Although exposure to conventional microplastics such as polyethylene terephthalate (PET) has been shown to elicit distinct developmental and morphological changes in soil amoebae [30], the specific interactions between amoebae and biodegradable microplastics remain virtually unexplored.

We hypothesize that PLA microplastics impair amoebae chemotaxis and predation, thereby reducing their efficiency in locating and preying on soil bacteria. To test the hypothesis, we amended natural soils with PLA microparticles to construct experimental micro-interfaces, and subsequently profiled shifts in microbial community composition and functional potential, with a particular emphasis on soil amoebae. In parallel, controlled laboratory assays were conducted to evaluate changes in amoebae chemotactic responses and predation dynamics. By systematically assessing how biodegradable PLA microplastics reshape the interactions between soil amoebae and their bacterial prey, this study provides insights into the dynamic impacts of biodegradable microplastics on soil microbial community structure and function.

## Materials and methods

### Microcosm setup and field incubation of polylactic acid microplastics

Experimental design is summarized in Fig. S1. The soil used in this study was sourced from Guangzhou, China (23°4′N, 113°22′E), with key physicochemical properties summarized in Table S1. The PLA microplastics, which were obtained from NatureWorks LLC (USA), were sterilized using UV radiation before their use in soil experiments. To maximize microbial interaction, 2 g of PLA microplastics (3 mm, 100 μm, and 10 μm) were uniformly distributed as thin films at 10 cm soil depth. The soil interaction volume for each experimental unit was defined as 10 × 10 × 10 cm. Within this volume, the applied contaminants reached a local concentration of 1500 mg/kg, integrating field mulch residues and soil PLA studies [31, 32]. Sterilized SiO_2_ particles (121°C, 20 min) of equal mass served as the inert control, following the established practice of employing glass beads to decouple microplastic-specific effects from general particle impacts [33]. All particles were encapsulated in 1200-mesh nylon bags to minimize degradation-induced volume changes [34]. Consequently, incubation was from 18 February to 19 March 2023. The temperature range during this period was 8°C to 29°C (Fig. S2). After incubation, soil samples were collected 5 cm from the PLA layers using a sterile coring device.

### DNA extraction, metagenomic sequencing, and bioinformatic analysis

Soil DNA was extracted using the Soil DNA Isolation Kit (MoBio) and quantified via Qubit 2.0 Fluorometer (Life Technologies, CA, USA). The DNA was then sequenced using 150-bp paired-end reads on a NovaSeq 6000 System (Illumina) at Novogene (Tianjin, China). Raw sequencing data were processed through Trimmomatic (version 0.36; default parameters). The structures of microbial communities were annotated via Kraken2 based on the standard_PF database [35]. Current databases only contain genomes of several model organisms, which may lead to the underestimation of protist diversity. Then, the clean data were assembled with MEGAHIT (default parameters, including -k-list with values 21, 29, 39, 59, 79, 99, 119, and 141; contigs < 1 kb were discarded) within the MetaWRAP pipeline (version 1.3.2) [36, 37]. The open reading frames of contigs were predicted by Prodigal (version 2.6.3, −p meta). Then, antibiotic resistance genes (ARGs), metal resistance genes (MRGs), and mobile genetic elements (MGEs) were annotated against SARG (v3-S), BacMet2 (v2.0), and a manually curated MGE database (2706 sequences) via DIAMOND (*E* ≤ 1e-5, query coverage ≥ 80%, and ≥ 75% amino acid identity) [38–40]. Gene abundances were normalized to Transcripts Per Million (TPM) using Salmon [41].

### Amoeba cultivation and polylactic acid exposure

Drawing from the field incubation results, we selected a representative soil amoeba for further investigation. Under a diet of soil bacterium *Klebsiella pneumoniae* KpGe, *D. discoideum* QS9 was grown from spores on SM/5 agar plates (2.0 g glucose, 2.0 g BactoPeptone (Oxoid), 2.0 g yeast extract (Oxoid), 0.2 g MgCl_2_, 1.9 g KH_2_PO_4_, 1.0 g K_2_HPO_4_, and 15.0 g agar per liter) at 21°C with light. A second generation of spores was used for all experiments [42, 43]. Informed by global background levels of PLA in agricultural soils and exposure concentrations from microplastic-amoebae studies [32, 44], we selected four environmentally relevant concentrations (50, 500, 1500, and 2500 mg/kg), encompassing conditions from routine practice to high-exposure scenarios [45, 46]. Prior to their use in exposure experiments, the polylactic acid microplastics (PLA MPs) were aseptically dispersed in sterile Milli-Q water and then ultrasonicated for 10 min at 21°C to disrupt loose aggregates.

For PLA exposure, amoeba-PLA MPs interaction-interfaces were established. Amoeba spores (2 × 10^5^) were mixed with 200 μL of *K. pneumoniae* KpGe suspensions (OD_600_ = 1.5) and subsequently mixed with 50 μL of PLA MPs (with particle sizes of 100 μm and 10 μm) suspensions at four concentrations on SM/5 agar plates. The plates were incubated at 21°C with light for 7 days. A control group, which received 50 μL of sterilized water devoid of PLA MPs, was also included.

For the soil microcosmic experiment, soil was sourced from Guangzhou, China (23°4′N, 113°22′E). Sterilized natural soil was utilized to construct PLA MP interfaces that more accurately reflect real-world conditions. The soil samples were processed by air-drying, sieving through a 2.0 mm mesh, and sterilization via autoclaving at 121°C for 20 min, repeated thrice. Sterilized soil (1.0 g per well) was uniformly added to 12-well plates and compacted by vibration to establish the microcosms. A mixture consisting of 6 × 10^5^ amoeba spores, 200 μL of *K. pneumoniae* KpGe suspension (OD_600_ = 30), and 50 μL of PLA microplastic suspensions at nine treatment groups (0 mg/kg, 10 μm, and 100 μm at 50, 500, 1500, and 2500 mg/kg, respectively) was combined and then introduced into each well. The experiment included four replicates for each treatment.

### Assessment of polylactic acid degradation by amoebae

To assess whether amoebae can ingest and degrade PLA MPs and their intermediates within the experimental timeframe in both soil and agar media, we established four parallel treatment groups to quantify PLA depletion in these matrices. A concentration of 1500 mg/kg PLA MPs with different diameters (3 mm, 100 μm, and 10 μm) was selected for all treatments to maximize the observable effects. The Control group consisted of PLA MPs dispersed on sterile agar. The Amoebae group involved PLA MPs co-cultured with amoebae and their bacterial prey. The Bacteria group controlled for bacterial effects by incubating PLA MPs with *K. pneumoniae* alone (OD_600_ = 1.5). The Soil group comprised PLA MPs retrieved from field burial experiments. Gel permeation chromatography (GPC) was performed on an Agilent 1260 Infinity II GPC/SEC system equipped with a refractive index detector and two PL Mixed-C columns connected in series (7.5 × 300 mm, 5 μm; guard column 7.5 × 50 mm, 5 μm). Chromatographic-grade tetrahydrofuran was used as the mobile phase at a flow rate of 1.0 ml/min, with the column oven maintained at 40°C. The system was calibrated against narrow-disperse PS standards. The injection volume was 50 μL. Pristine and amoeba-exposed PLA particles (SM/5) were analyzed by GPC.

The surface morphology of the PLA MPs after biological treatments was firstly examined using a cryo-focused ion beam scanning electron microscope (cryo-FIB/SEM, Thermo Fisher Scientific Aquilos 2). Subsequently, Fourier Transform Infrared (FTIR) spectroscopy was employed to investigate the changes of PLA MPs after various biological treatments. Prior to analysis, the pellets were collected from each treatment group, thoroughly rinsed with deionized water to remove residual biomass, blotted dry with filter paper, and then air-dried at room temperature to a constant weight. The infrared spectra were acquired using a Thermo Fisher Scientific Nicolet iN10 spectrometer equipped with an Attenuated Total Reflectance accessory. For each sample, the spectrum was recorded in the range of 4000–400 cm^−1^ with a spectral resolution of 4 cm^−1^ by averaging 32 consecutive scans to ensure an adequate signal-to-noise ratio.

### Chemotaxis and predation assays

Amoebae (10^5^ spores) were inoculated on the left side of a predefined midline. On the right side, a droplet containing a mixture of 10 μL of *K. pneumoniae* KpGe suspension (OD_600_ = 1.5) and PLA MP suspension was applied. The two populations were symmetrically aligned along the midline with a center-to-center distance of 1.2 cm. PLA MPs were tested at concentrations of 0, 50, 500, 1500, and 2500 mg/kg and at particle sizes of 10 and 100 μm. Amoebae traversing the midline within the field of view on SM5 plates were quantified after 36 h under a 4× microscope objective (Echo Revolve) [43, 47, 48]. To specifically assess chemotaxis, independent assays were conducted on non-nutrient agar with PLA MPs selectively added to either the spore or the bacterial population. After 7 days, fruiting bodies were collected from the bacterial side to determine the total spores.

For the non-nutrient experiment, we utilized non-nutrient agar plates (2.2 g KH_2_PO_4_, 0.7 g K_2_HPO_4_, and 15.0 g agar per liter) to avoid bacterial growth, constructing a PLA MP interface for directly observing the effects on amoebae. 2 × 10^5^ amoebae spores, 200 μL of *K. pneumoniae* KpGe suspension (OD_600_ = 30), and 50 μL of PLA microplastic suspensions at pre-set concentrations (0, 50, 500, 1500, and 2500 mg/kg) were mixed on non-nutrient agar plates and cultured for 7 days with light at 21°C. Additional plates received a single 3 mm PLA at the center of the microbial surface. After 7 days of cultivation, both the stalk lengths and the sorus diameters arising proximal to the PLA MPs were quantified [43]. Multiple 3 mm PLA particles were also placed on this interface, and a 5 cm agar strip was excised for photographic documentation of morphological changes in amoebae.

Predation assays were also created on non-nutrient agar plates. A mixture of 2 × 10^5^ amoeba spores and 200 μL of *K. pneumoniae* KpGe suspension (OD_600_ = 30) was spread evenly across the entire plate to create a microbial surface. On the left side of the plate’s midline, 1.0 g of microplastics was applied (PLA MPs of varying sizes: 3 mm, 100 μm, 10 μm, and SiO_2_ for control). The right side of the plate’s midline is left untreated. After incubation at 21°C with light for 36 h, the behavioral dynamics of amoebae at the midline boundary were observed. After 7 days, a 5 cm wide agar strip was cut from the center of each plate, and the distance between the closest fruiting bodies to the boundary was recorded.

In subsequent experiments, on the SM/5 and the non-nutrient agar plates, 2 × 10^5^ amoeba spores and 200 μL of *K. pneumoniae* KpGe suspension (OD_600_ = 30) were mixed, and PLA microplastics of 3 mm in size, along with glass beads as controls, were gently placed on the agar plates.

### Indirect interaction assays

Multi-faceted imaging approach was employed to investigate the temporal effects of PLA MPs on amoeba growth and aggregation. Specifically, on SM/5 plates, time-lapse imaging quantified the predatory activity proximal to the 3 mm PLA microparticles at 24, 36, and 48 h. Separately, on blank SM/5 plates, amoeba spores were pre-mixed with bacteria to form two populations with a center-to-center distance of 2.4 cm, and 3 mm PLA particles and glass beads were respectively placed at the left population. Concurrently, stereomicroscopy coupled with bright-field microscopy resolved migration patterns. The specific inhibition of bacteria (Table S2) that can be preyed upon by amoebae by PLA MPs was quantified by inhibition zone measurement and agar plate response. And the images were captured using a Canon camera (EOS 800D) 10 cm above the bottom surface of the microplastics. Separately, single and multiple 3 mm PLA particles were placed on this interface for imaging documentation. On this basis, five non-biodegradable microplastics (PVC, PA, PET, PE, and PP) were employed to validate predator avoidance behaviors, all maintained at a 3 mm particle size to serve as controls. Details are provided in the supplementary materials.

### Fitness and morphometric analyses

For treatments requiring spore collection on Day 7, we collected the amoeba spores by flooding the plates with 2 ml of KK2 buffer containing 0.1% NP-40 and transferred the suspension to a 2 ml centrifuge tube. For amoebae cultivating in the soil microcosms, fruiting bodies were harvested using a sterile inoculating loop, and then placed into 1 ml of KK2 buffer. The production of total spores was used as an indicator of amoebae fitness. For morphometric analysis, the length of the stalk and the diameter of the sorus were measured [43].

### Statistical analysis

Statistical analyses of the microbial community data were performed using R software (version 4.3.2) and the *vegan* package. Shannon Index for Bacteria, Archaea, Eukaryota, and Viruses were compared across four treatments (CK, 3 mm PLA, 100 μm PLA, and 10 μm PLA) using one-way ANOVA followed by Tukey’s *post hoc* test. For spatial distribution patterns, permutational multivariate ANOVA (PERMANOVA) followed by principal coordinate analysis (PCoA) was applied to assess variance in microbial community composition. Multivariate dispersion patterns (MDS plots) and univariate trait distributions (ARG and MRG abundances) were compared across four treatments. ANOSIM was applied to assess community structuring. All boxplot comparisons and related diagrams were similarly analyzed using ANOVA to evaluate treatment effects. Statistical significance was determined at *P* < .05, with Bonferroni correction applied for multiple comparisons.

## Results

### Polylactic acid microplastics restructure soil microbial networks and inhibit predatory protists

**Figure 1:**
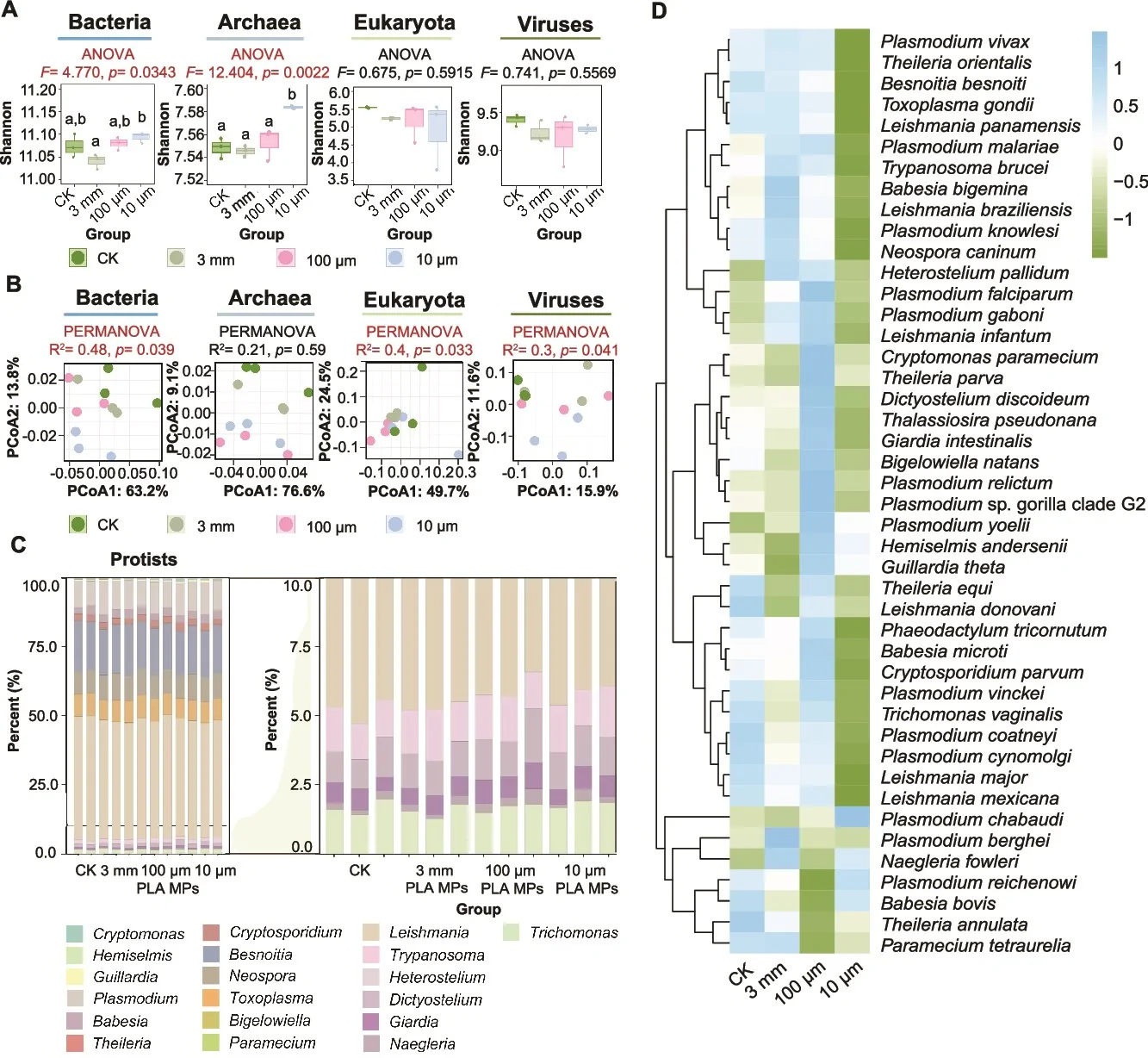
Microbial community profiling of the treatment group. (A) Alpha diversity comparison across bacterial, archaeal, protistan, and viral communities using Shannon Index. (B) PCoA based on Bray–Curtis dissimilarities, with treatment effects assessed by permutational multivariate analysis of variance (PERMANOVA). (C) Relative abundance distribution of protistan taxa at phylum level (100% stacked bars), with expanded inset (scale ×10) depicting *D. discoideum* population dynamics. (D) Species-level compositional changes of soil protists under PLA MPs insertion revealed by heatmap. Rows were normalized to Z-scores based on relative abundances, highlighting deviations relative to each species’ mean abundance. The color scale is consistent across all columns.

Field incubations revealed apparent shifts in microbial diversity across all four domains (Fig. 1, Fig. S3). Bacterial Shannon Index varied (Fig. 1A), with ANOVA revealing significant differences (*F_3,12_* = 4.8, *P* = .034), whereas archaeal communities exhibited even stronger treatment responses (*F_3,12_* = 12.4, *P* = .002). A consistent trend of perturbation was observed across multiple alpha-diversity analyses (Fig. S4, Fig. S5). Beta diversity analyses further stratified these responses (Fig. 1B): archaeal communities remained stable (PERMANOVA, *R*^2^ = 0.21, *P* = .59), whereas bacterial (*R*^2^ = 0.48, *P* = .039), eukaryotic (*R*^2^ = 0.40, *P* = .033), and viral (*R*^2^ = 0.30, *P* = .041) exhibited significant spatial differentiation driven by PLA particle (PCoA1 variance explained).

Protists dominated eukaryotic communities (19 genera identified), with PLA exposure altering genus-level distribution (Fig. 1C). Algae (including *Cryptomonas, Hemiselmis,* and *Guillardia*, all belonging to the *Cryptophyta*), parasitic protists (including *Leishmania, Trichomonas,* and 10 others), and predatory protozoa (*Dictyostelium, Heterostelium,* and *Paramecium*), as well as mixotrophic protists (*Bigelowiella*) were identified. Among the diverse protists, we focused on heterotrophic species with potential interactions with soil bacteria. The PLA layer induced a downward trend in the proportion of protists among eukaryotes (Fig. 1D, Fig. S6), which implied a redistribution among various predatory protists.

### Polylactic acid microplastics affect the metabolic potential of soil microbiome and enrich antibiotic resistance genes

**Figure 2:**
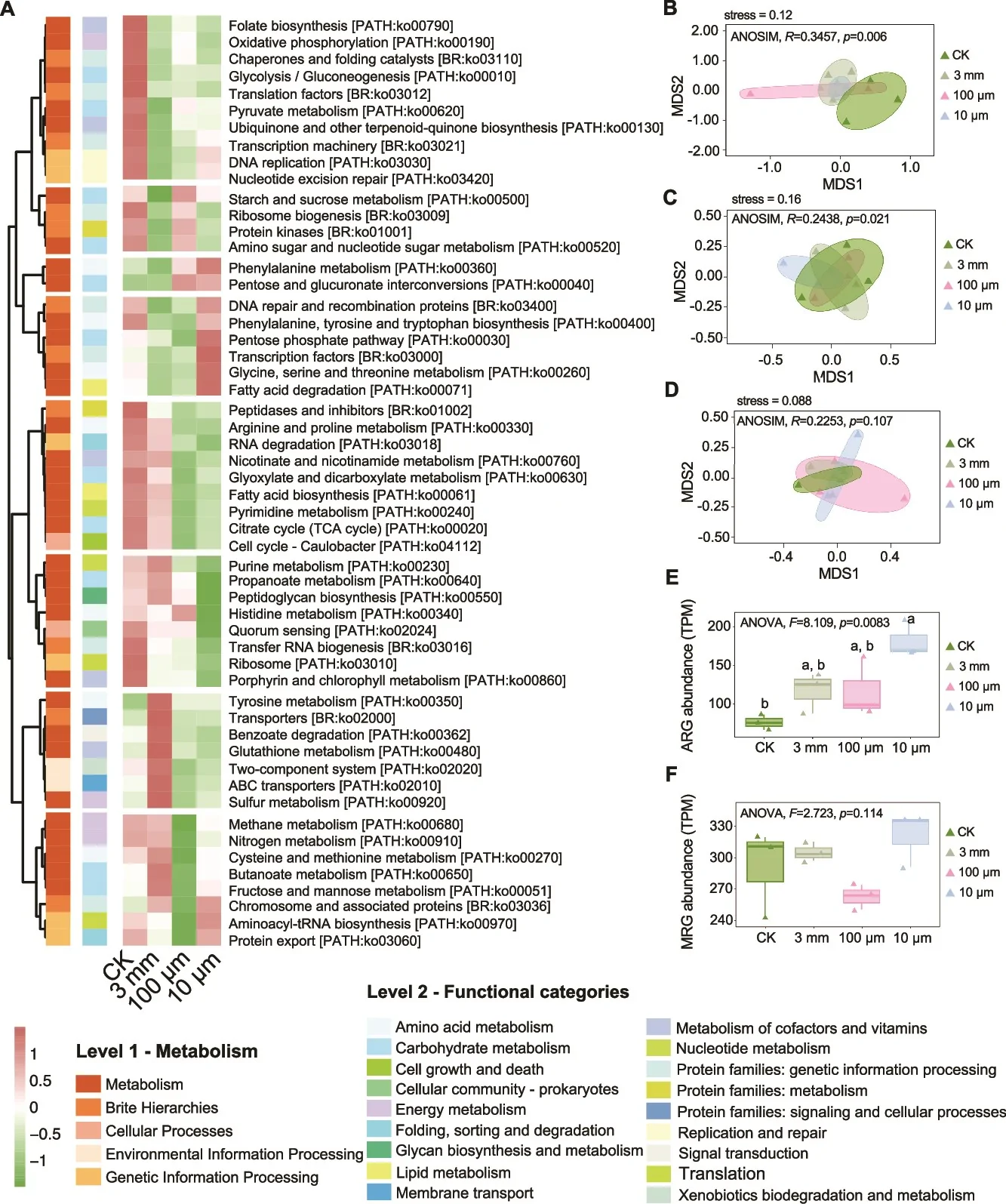
Impact of PLA treatment on antibiotic resistance determinants and metabolic gene abundance. (A) Heatmap of gene abundance profiles across four PLA treatment groups, categorized by KEGG metabolic pathway. (B–D) Non-metric multidimensional scaling (NMDS) analysis of ARGs, MGEs, and resistance-related genes (MRG) under different treatments. (E) Comparative analysis of ARGs abundance across treatments. (F) Comparative analysis of resistance-related genes (MRG) abundance across treatments.

To explore potential functional responses, analysis of metabolic pathway profiles revealed distinct patterns across the four PLA treatment groups. Hierarchical clustering of KEGG pathways further identified clusters of pathways exhibiting correlated functional potentials across the four PLA treatments (Fig. 2A). A gradient color scale shows covariation patterns in pathway abundances under PLA exposure. Specifically, multiple pathways, including glycolysis/gluconeogenesis and transcription machinery, exhibited lower relative abundances in specific treatment groups, whereas phenylalanine metabolism and fatty acid degradation showed higher relative abundances (10 μm PLA group). Hierarchical clustering grouped tyrosine metabolism and sulfur metabolism with environmental information processing pathways (such as ABC transporters and the two-component system), suggesting coordinated stress responses to PLA. The reduction in gene abundance associated with core energy metabolism pathways (oxidative phosphorylation) implies a possible diminishment of the capacity for energy metabolism in response to PLA.

ARG profiles exhibited the strongest treatment divergence (*R* = 0.35, *P* = .006, stress = 0.12, Fig. 2B). MRG patterns showed intermediate differentiation (*R* = 0.24, *P* = .021, stress = 0.16, Fig. 2C), where 10 μm MPs grouped separately from CK but maintained partial overlap with larger MPs. In contrast, MGE distributions demonstrated nonsignificant structural shifts (*R* = 0.23, *P* = .11, stress = 0.088, Fig. 2D), with substantial overlap among group ellipses. Quantitative analysis revealed 10 μm PLA MP treatment showed higher ARG abundance than CK (*F* = 8.1, *P* = .0083, Fig. 2E), whereas total TPM-normalized MRG abundance did not differ significantly among treatments (*F* = 2.7, *P* = .11, Fig. 2F).

### Polylactic acid microplastics inhibit the growth and morphology of amoebae

To further explore the effect of PLA addition on amoeba fitness, we utilized *Dictyostelium discoideum* (a terrestrial model protist), a representative amoeba species identified from field assay as being responsive to PLA MP exposure, for additional experimental validation.

**Figure 3:**
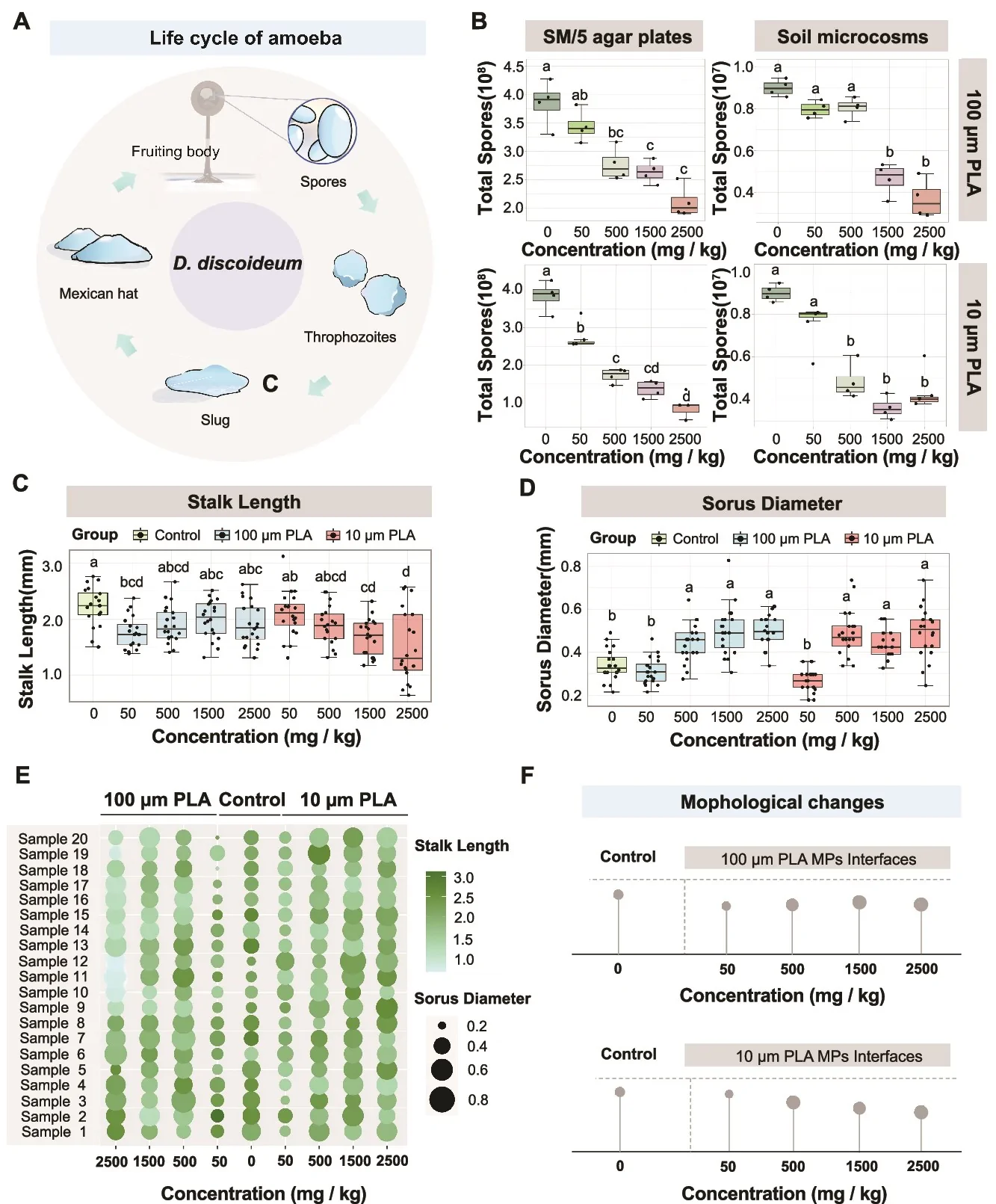
PLA MPs inhibit the growth and morphology of *D. discoideum*. (A) Life cycle of *Dictyostelium discoideum*. Key developmental stages include spores, trophozoites, slug, and Mexican hat, with aggregation representing a critical transition process. (B) Spore production of *D. discoideum* QS9 under two microplastic conditions: 100 μm PLA MPs and 10 μm PLA MPs, cultivated on SM/5 agar plates versus soil microcosms. (C) Change in stalk length after exposure to different concentrations of PLA MPs. (D) Change in sorus diameter after exposure to different concentrations of PLA MPs. (E) Visualization of sorus diameter and stalk length in fruiting bodies exposed to PLA MPs. (F) Morphometric comparisons of fruiting body architectures based on mean values.

At the microbial-microplastic interfaces where three key interactors (including amoeba, soil bacteria, and PLA MPs) converge, we assessed amoeba’s fitness and evaluated the fitness costs of PLA MPs exposure*. Dictyostelium discoideum* completed its life cycle on amoeba-PLA interfaces (Fig. 3A). Compared to the control, a significant reduction of total spores and alteration of fruiting body morphology in *D. discoideum* were observed with elevated PLA microplastic concentrations compared to controls (Fig. 3B–D, *P* < .05, ANOVA). Enlarged sorus diameters and shortened stalk lengths were also observed after exposure to elevated concentrations of PLA MPs (Fig. 3C–F, *P* < .05, ANOVA). Soil microcosm experiments corroborated these findings (Fig. S7), demonstrating a consistent inhibition of *D. discoideum* fitness with increasing PLA concentrations, with total spores declining by more than 50% at 2500 mg/kg (Fig. 3B). Microbe–microplastic interactions were also evident within the complex soil structure.

### Effect of amoebae on the degradation of polylactic acid microplastics

**Figure 4:**
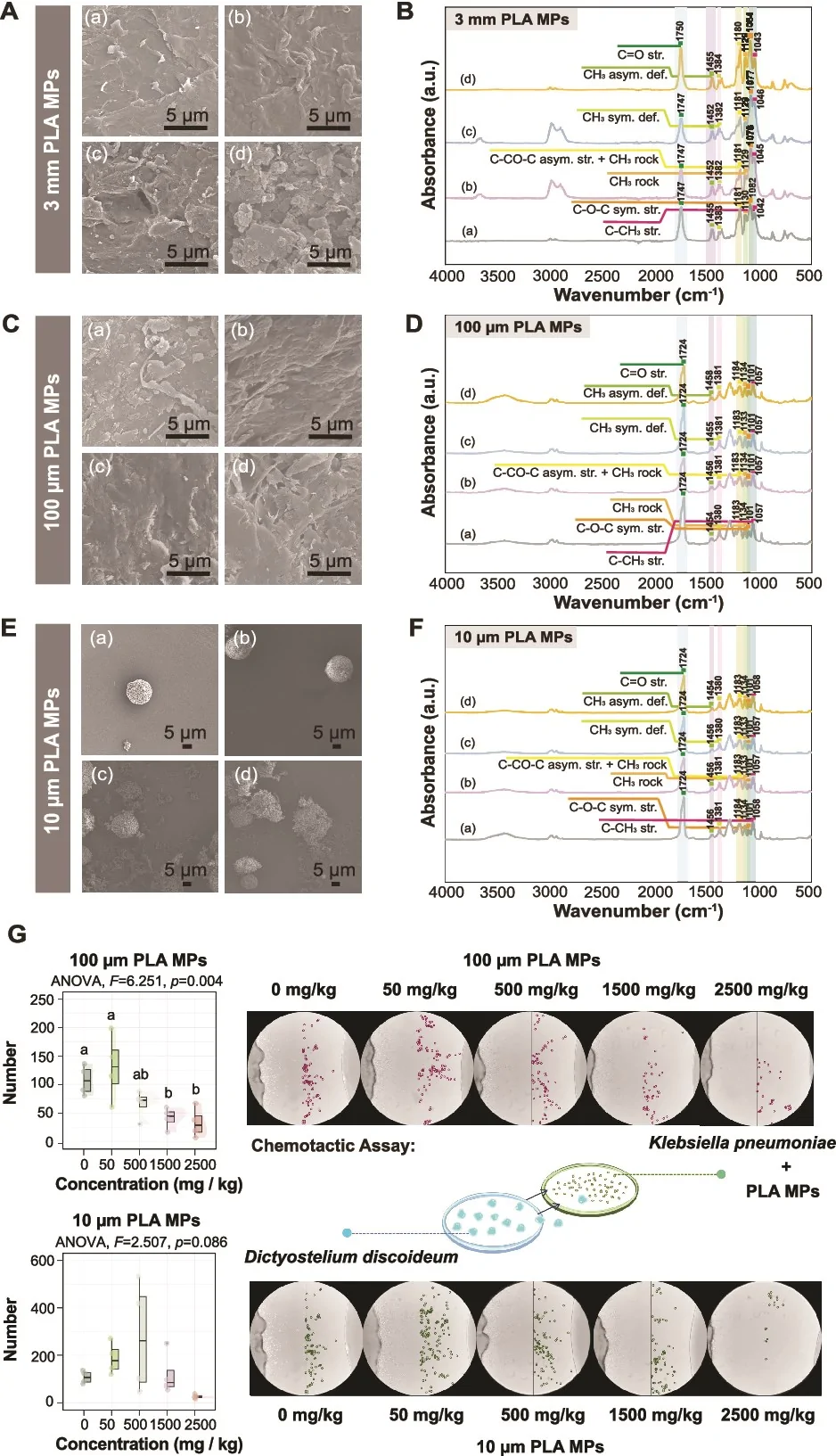
Bidirectional interactions between amoebae and PLA MPs and their degradation dynamics and chemotactic responses. (A)–(F) show the surface morphology and chemical functional group changes of PLA microplastics under four treatment conditions. Representative scanning electron microscopy (SEM) images of PLA MPs with diameters of (A) 3 mm, (C) 100 μm, and (E) 10 μm. FTIR spectra of PLA microplastics after different biological treatments with a diameter of (B) 3 mm, (D) 100 μm, and (F) 10 μm. For each panel, the spectra correspond to the following treatments: (a) dispersion in sterile medium (Control), (b) co-culture with bacteria (Bacteria), (c) co-culture with an amoeba-bacteria system (Amoeba), and (d) soil incubation (Soil). All spectra were recorded in absorbance mode using an FTIR spectrometer. All key characteristic peaks corresponding to PLA are highlighted [49]. (G) Chemotaxis experiments (counting the number of amoebae crossing the midpoint towards *K. pneumoniae* mixed with 100 μm PLA MPs, and *K. pneumoniae* mixed with 10 μm PLA MPs), photo records of the counts, and schematic diagrams of the experiments. The irregularity of the droplet edge stems from the uneven distribution of microbial growth on the SM/5 agar plate. Motile cells are highlighted with circular annotations.

We examined the bidirectional interactions between amoebae and PLA MPs. Following co-culture with amoebae, GPC revealed molecular-level degradation of PLA. *M*_n_ decreased from 66 663 to 52 246 g/mol (−21.7%), *M*_w_ from 99 093 to 87 006 g/mol (−12.2%), and *M*_z_ from 145 591 to 132 094 g/mol (−9.3%) (Table S3), indicating random chain scission (Fig. S8). For each particle size, SEM analysis indicated incipient fragmentation of 3 mm, 100 μm, and 10 μm PLA MPs by amoebae and their bacterial prey (Fig. 4A, C, E). Overall, characteristic IR peaks of PLA across the four treatments showed only minor shifts, with representative assignments including C=O and C–O–C (Fig. 4B, D, F). Taken together, these findings demonstrate that *D. discoideum* promotes the depolymerization and physical breakdown of PLA microplastics. However, no substantial changes in the chemical functional groups of the PLA MPs were detected.

### Polylactic acid microplastics affect chemotaxis and predation in amoebae

In the chemotaxis experiments, *D. discoideum* QS9 exhibited chemotactic responsiveness toward bacterial food (*K. pneumoniae* KpGe mixed with PLA MPs) in all treatment groups by 36 h (Fig. 4G). However, increasing PLA MP concentrations (ranging from 0 to 2500 mg/kg) caused a significant decline in the number of amoebae reaching the midpoint (*P* < .05, ANOVA), signifying an impaired chemotactic ability (Fig. 4G).

**Figure 5:**
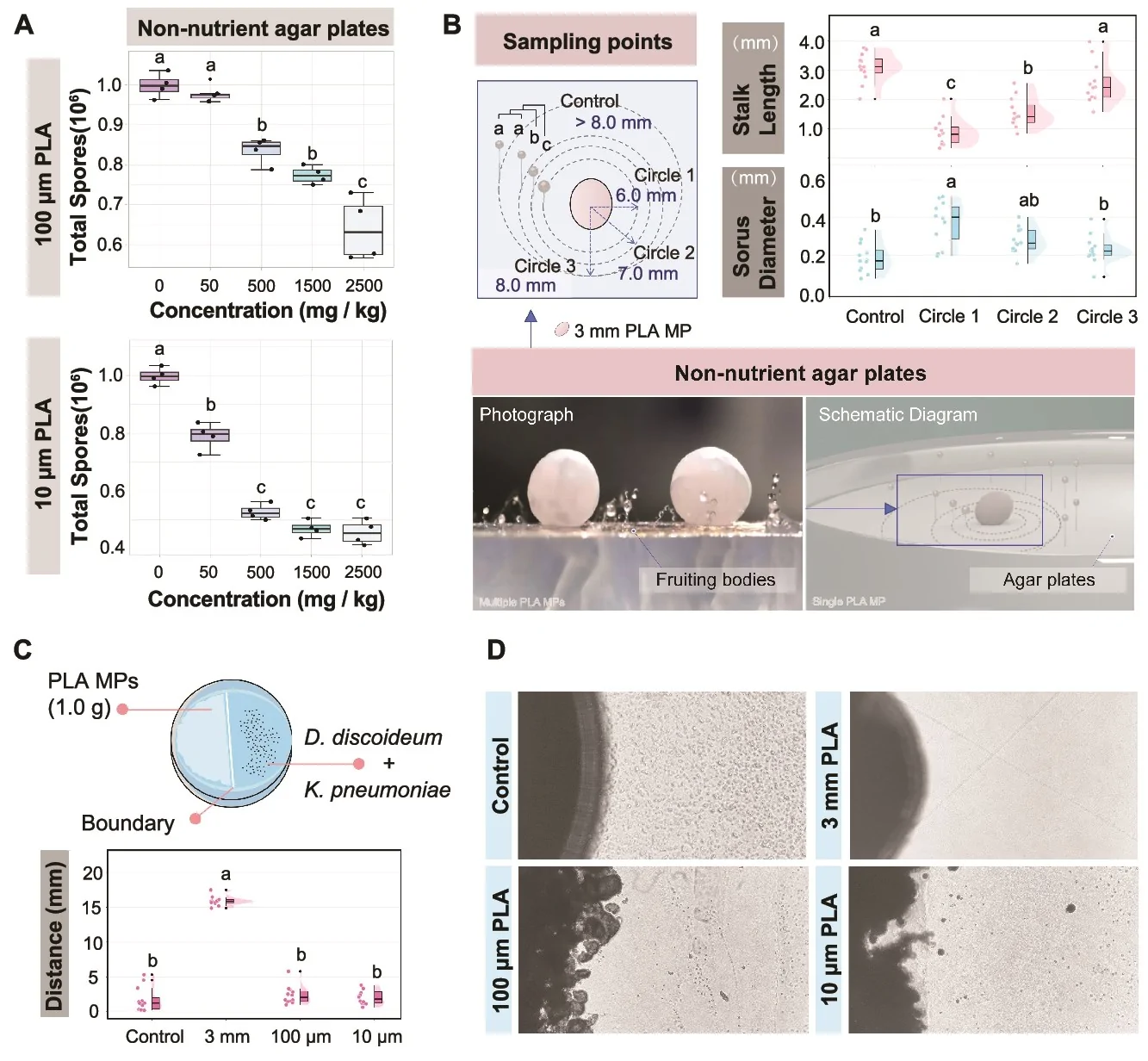
PLA MPs affect predation in *D. discoideum*. (A) Total spores on non-nutritive microplastic surfaces, cultivated for 7 days on 100 μm and 10 μm PLA MPs. (B) Spatial colonization patterns around 3 mm PLA MPs: (i) Planar schematic showing three sampling zones (6, 7, 8 mm from center) in quadruplicate experiments; (ii) stalk length/sorus diameter distributions via cloud rain plots; (iii) Photograph and 3D schematic diagram using Blender v3.6.5. (C) Microbial colonization distance (μm) from microplastic boundaries in microbial–microplastic interaction-interfaces. (D) Species interaction dynamics: *D. discoideum* and bacterial colonization patterns near boundaries within 24 h.

Additional chemotaxis assays were conducted to investigate this effect (Fig. S9). The results, which include the experimental setup (Fig. S10) and statistical analysis (Fig. S11), consistently showed that PLA MPs impaired the complete chemotaxis and predation of amoeba. Such chemotactic deficits disrupt the foundational predator–prey dynamics, potentially destabilizing soil microbial networks. The amoeba–PLA interactions were also established on non-nutritive agar plates (Fig. S12), which further corroborated the direct impact of PLA MPs on *D. discoideum* QS9 with a marked reduction in total spore production (Fig. 5A, *P* < .05, ANOVA). At specific radial distances (6–8 mm and >8 mm) from the 3 mm PLA microplastic interaction-interfaces, we analyzed fruiting body development and observed statistically significant morphometric differences (Fig. 5B, *P* < .05, ANOVA). Specifically, stalk lengths exhibited a 41.2% reduction, whereas sorus diameters increased by 23.6% compared to controls. These morphological deviations correlated strongly with proximity to the PLA interaction-interfaces, with more pronounced alterations observed in proximity to the microplastic boundary (Fig. 5B, *P* < .05, ANOVA).

In complementary experiments using non-nutrient agar plates, we confined equal masses (1.0 g) of millimeter-scale (3 mm) and micrometer-scale (100 μm and 10 μm) PLA MPs to opposite sides of the plate. Microscopic observation revealed a clear boundary-mediated behavioral response: *D. discoideum* cells exhibited a 3.5-fold increase in avoidance distance (8.2 ± 1.5 mm) near the 3 mm PLA interaction-interfaces relative to 100 μm PLA MPs (2.3 ± 0.7 mm, *P* < .05, ANOVA, Fig. 5C–D). This boundary avoidance was size-selective, with millimeter-scale PLA eliciting stronger repulsion than micrometer-scale particles (*P* < .05, ANOVA; Fig. 5C). Furthermore, multi-particle 3 mm PLA aggregates induced repulsion at greater distances compared to single particles, suggesting emergent interfacial interactions.

### Polylactic acid microplastics indirectly impact amoebae by altering food bacteria

**Figure 6:**
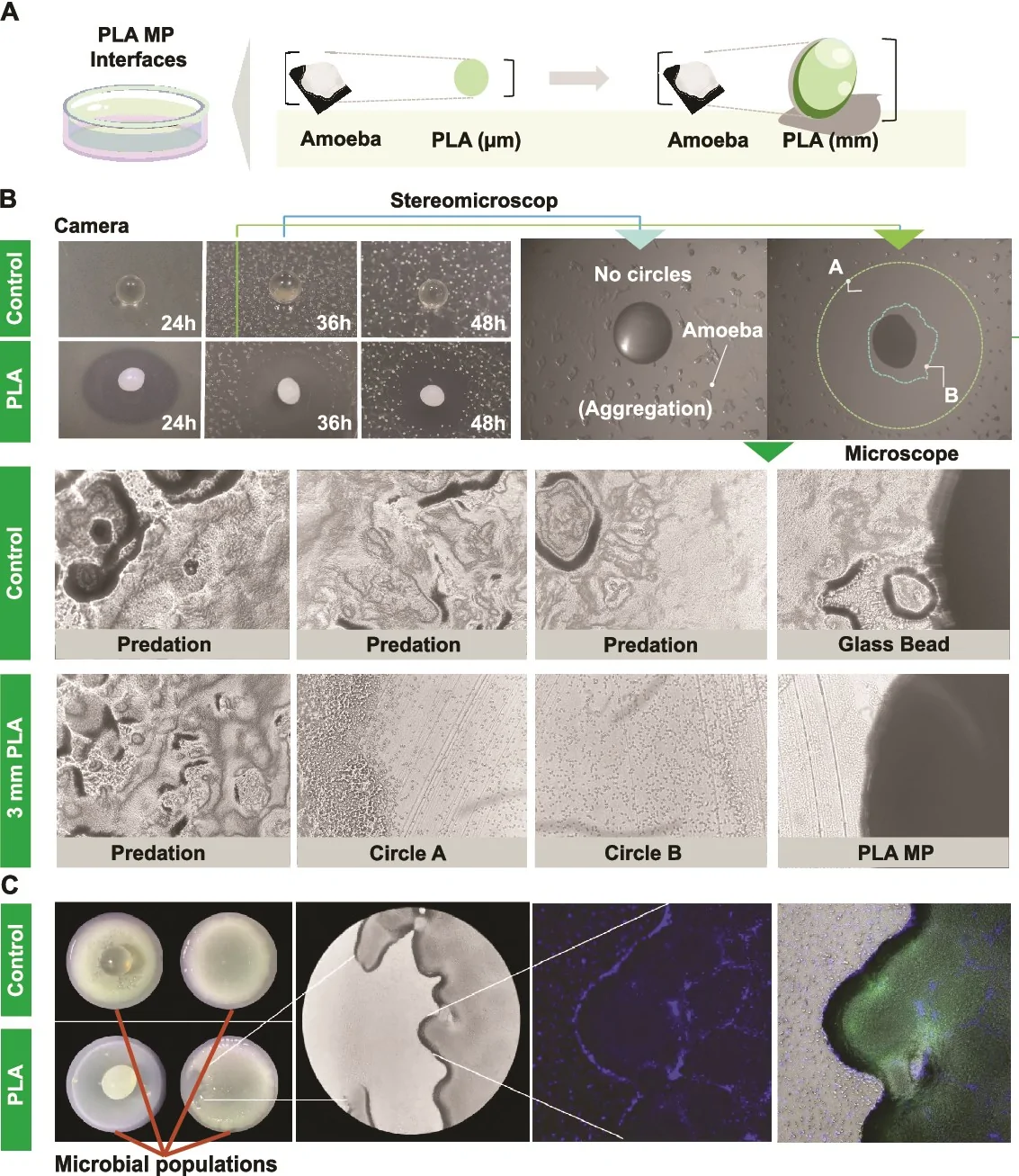
PLA MPs indirectly alter *D. discoideum* by altering food bacteria. (A) Proposed indirect mechanism of PLA MPs on amoeba–prey interactions. (B) Temporal evidence of prey alteration and its impact on amoeba growth. Time-lapse photography: Quantification of colony diameter (μm) at 24, 36, and 48 h. Stereomicroscopy: Capture of amoeboid migration patterns (A/B rings) during the aggregation phase (36 h). Bright-field microscopy: High-resolution imaging of ring formation structures. (C) Macroscopic, microscopic, and fluorescence imaging reveal PLA-induced bacterial inhibition zones and enhanced amoeboid aggregation.

When scaled to millimeter dimensions, the introduction of 3 mm PLA microplastics created a magnified biophysical interaction-interfaces between amoeba QS9 and food bacteria KpGe (Fig. 6A). Time-lapse photography demonstrated distinct inhibitory effects on both amoeba and food bacteria surrounding 3 mm PLA MPs, revealing both bacterial exclusion and amoeboid navigation failure (Fig. 6B). 100% of fruiting bodies circumvented PLA MPs by 48 h (versus 0% for glass beads).

Cross-scale spatial disruption was also extended beyond direct contact, inducing systemic growth deficits in adjacent microbial populations. Two microbial populations, spaced 1.2 cm apart on non-nutritive agar plates without microplastics, served as spatially segregated units under identical conditions. After 36 h incubation, control populations with glass beads showed no interaction. However, PLA exposure directly inhibited adjacent microplastic-free microbial populations, with growth deficits reaching eight times the PLA radius. Fluorescence microscopy of GFP-labeled *K. pneumoniae* KpGe also revealed no bacterial fluorescence in growth-deficient zones (Fig. 6C, Fig. S13).

**Figure 7:**
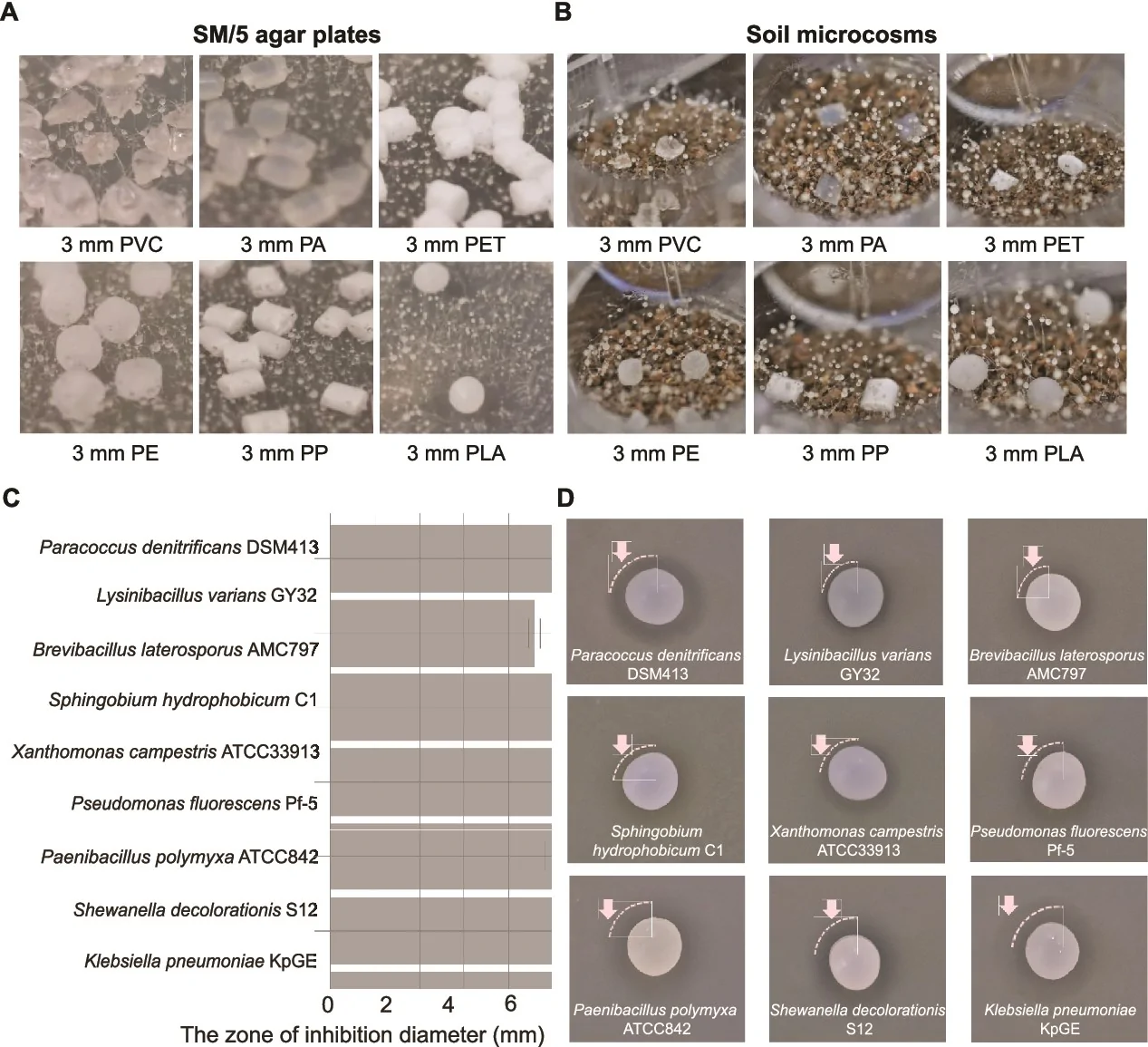
Impact of 3 mm microplastics on soil protist-bacterial interactions. (A) 3 mm microplastics (PVC, PA, PET, PE, PP, and PLA) were incubated with *D. discoideum* QS9 on SM/5 agar plates. (B) 3 mm microplastics (PVC, PA, PET, PE, PP, and PLA) were incubated with *D. discoideum* QS9 in soil microcosms. (C) The diameters of inhibition zones measured for multiple soil bacteria susceptible to amoebae predation after 5-day incubation under 3 mm PLA MPs stress. (D) Response of multiple soil bacteria to PLA exposure on SM/5 agar plates.

Contrary to traditional microplastics, *D. discoideum* QS9 exhibited specific avoidance behavior exclusively to 3 mm PLA MPs (Fig. 7A, Figs S14–S16). PE, polyamide (PA), PP, PVC, and PET MPs at equivalent sizes (3 mm) showed no significant repulsion effect on amoeboid migration or bacterial colonization (Fig. 7B, Fig. S17). We further investigated the responses of multiple soil bacterial species that serve as prey for amoebae to the presence of 3 mm PLA MPs. Clear inhibition zones were observed, indicating antibacterial effects (Fig. 7C–D). Comparable inhibition zones formed upon the addition of monomeric lactic acid (pH = 2 and pH = 3) to the agar plates (Fig. S18). The results also indicate that PLA MPs fragment the bacterial network (Fig. S19). This long-range biophysical coupling demonstrates that PLA MPs reconfigure microbial interactions beyond direct contact, inducing a continuum of responses to PLA MPs spanning microscale chemotaxis to macroscale spatial exclusion.

## Discussion

Adverse effects of biodegradable PLA microplastics on soil microbial communities are well recognized, but the functional responses of predatory protists have received little attention. Our study investigates the amoeba-bacteria-PLA interface, providing a protistan perspective to trace ecological impacts from cellular to community levels. We demonstrate that PLA microplastics reshape the microbial community structure (Fig. 1) and functional potential (Fig. 2), thereby mechanistically supporting growing concerns about their environmental safety. PLA alters the predator–prey dynamics between soil amoebae and bacteria, shifting the energy investment and foraging strategies of this key predator and then exerting an upward control over the microbial metabolic network. This work is the first to elucidate the ecological consequences of PLA microplastics from the standpoint of protistan predation, thereby filling a critical knowledge gap.

Central to this gap is the recognition that although PLA fosters a unique plastisphere capable of restructuring prokaryotic communities [33, 50, 51], the resulting ecological ripple effects remain poorly understood. Prior work indicates that such perturbations can reduce soil microbial diversity and simplify rhizosphere networks, occasionally exceeding those of conventional PVC [16, 52]. Echoing these observations, PLA microplastics directly impair model predatory amoebae by reducing total spores and compromising chemotaxis (Figs 3, 4), inflicting quantifiable fitness costs that disrupt predatory efficiency and spatial colonization. These costs carry profound ecological implications for soil ecosystems, directly reflecting the stress imposed on predatory microorganisms. This inhibition is closely linked to the release of lactic acid oligomers during PLA weathering, which drives localized pH declines [16, 53]. Previous investigations into this mechanism have primarily focused on the direct alteration of microbial community function by PLA, or on the selective pressure exerted on surface-colonizing microbes within the PLA plastisphere [33]. Our results provide a more precise mechanistic explanation that PLA can simultaneously act on different trophic levels within the food web, exerting varying degrees of negative effects on both bacteria and their protistan predators, thereby disrupting the critical interactions that underpins ecosystem function. Even a single 3 mm PLA microplastic, whose volume vastly exceeds that of amoeba spores, creates a distinct and disproportionate stressor, and the presence of multiple particles amplifies this effect (Fig. 5). This focus on a model amoeba and pure PLA provides a crucial mechanistic baseline, collectively establishing 1500 mg/kg as a critical threshold for PLA impact under our experimental conditions. This baseline is particularly important given that potential risks of PLA are a known concern in intensive agricultural systems, where PLA mulch films are widely applied [32, 54]. By bridging laboratory assays with environmental relevance, our exposure experiment identified this key effect threshold. Although the higher concentrations used may represent localized “hotspot” scenarios, they are critical for understanding potential risks in intensive systems and elucidating the maximum toxic potential [13, 44, 55]. Such high-exposure scenarios can be effectively mitigated through improved agricultural management. This includes selecting PLA products with degradation cycles tailored to local climates and crop needs, and planning their safe service life according to cropping patterns whereas ensuring thorough post-harvest recovery. Furthermore, monitoring should extend beyond crop yield and plastic integrity to include key soil predators like amoebae, focusing on the predatory dynamics at the PLA-amoeba-bacteria interface. Our results thus reveal not only the impairment of soil microorganisms by PLA microplastics but also a hidden ecological cost associated with the increasing reliance on BDPs.

The inhibition of key microbial predators like amoebae (Fig. 6) should not be viewed as an isolated toxicological endpoint. Existing research indicates that the impact of PLA MPs on the soil ecosystem is systemic, involving not only direct interference with key biogeochemical cycles such as nitrogen and phosphorus [21, 56], but also the integration of its degradation products as an unconventional carbon source into microbial metabolic networks, driving alterations in metabolic potential [57, 58]. Our study provides direct mechanistic evidence for this, confirming reduced relative abundances of core energy metabolism pathways and elevated relative abundances of peripheral catabolism pathways. This indicates a fundamental shift in microbial carbon and energy allocation patterns, aligning with previous findings that PLA perturbs symbiotic metabolic exchanges, such as hydrogenotrophic methanogenesis [59]. This metabolic shift is part of a coordinated community response to PLA exposure. By exerting multifaceted stressors on both amoebae and their bacterial prey (Fig. 7), PLA MPs compromise the functional integrity of the soil microbial network, challenging the long-standing assumption that BDPs inherently ensure environmental safety. The predatory interactions of soil protists like amoebae offer a microcosm of the intricate balance that underpins soil food webs [60, 61]. This dynamic interplay is critical for maintaining ecosystem equilibrium and resilience. Translating these findings to human health contexts is fraught with complexity but warrants scrutiny. PLA can be enzymatically degraded into oligomeric nanoparticles within the gastrointestinal tract, which not only integrate into host carbon metabolism but also trigger specific inflammatory pathways [58, 62]. Future work should test whether the observed functional perturbations in amoebae translate to similar dysfunction in macrophages exposed to PLA degradation products and compare the distinct ecotoxicological profiles of biodegradable versus conventional microplastics. Furthermore, the distinct ecotoxicological profile of biodegradable microplastics, including their enhanced potential for pollutant adsorption, must be considered in biomedical risk assessment.

The findings presented in this study provide a transferable, integrative framework for systematically assessing the complex environmental risks of biodegradable PLA plastics. Given the rapid increase in PLA production and application [63], its risks under specific environmental exposures warrant closer scrutiny. In agriculture, mulch films are often formulated as blends with additives, with PLA-PBAT being a common composite [64, 65]. The fate and ecological impacts of such complex formulations can differ substantially from those of pure PLA due to interactions between polymers [53, 66], which underscores the need for future field trials across diverse agroecosystems to complement controlled studies. As a first step, the amoeba-PLA interface established here using pure PLA provides a fundamental reference point against which the effects of blending components can be rigorously benchmarked. Our findings point to three critical imperatives. First, amoebae should be adopted as sentinel bioindicators to operationalize soil health monitoring. Second, in the context of risk assessment, this work offers a new perspective that moves beyond traditional chemical endpoints toward more ecologically relevant guidelines for agricultural plastic use. Third, the pure-PLA baseline established here serves as a reference for interrogating the behavior of complex commercial blends *in situ*. Therefore, by dissecting the amoeba-bacteria-PLA interface, this study transcends the conventional view of PLA microplastics as passive pollutants and provides a transferable paradigm for understanding the multifaceted impacts of materials in complex ecosystems.

## Supplementary Material

Supplementary_information_final_wrag237

## Data Availability

Raw sequence data have been submitted to NCBI Sequence Read Archives (SRA) with the accession number PRJNA1285071.
