# Supplementary material for: Biodegradable polylactic acid microplastics rewire the soil food web by impeding amoebae predation
Source: ISME J. 2026 Sep 12;20(1):wrag237. doi: 10.1093/ismejo/wrag237 (PMC13615528; doi:10.1093/ismejo/wrag237)
Supplement: Supplementary file 1 [file supplementary_information_final_wrag237.docx]

*Supplementary information*

**Biodegradable polylactic acid microplastics rewire the soil food web by impeding amoebae predation**

Lin Zhang^#^, Fei Liu^#^, Lu Ma, Min Zhou, Yuanchen Zhao, Yiyan Yin, Bo Wu*, Longfei Shu*

School of Environmental Science and Engineering, Southern Marine Science and Engineering Guangdong Laboratory (Zhuhai), Guangdong Provincial Key Laboratory of Environmental Pollution Control and Remediation Technology, State Key Laboratory for Biocontrol, Sun Yat-sen University, Guangzhou 510006, China

***Correspondences**

Prof. Longfei Shu ([shulf@mail.sysu.edu.cn](mailto:shulf@mail.sysu.edu.cn))

School of Environmental Science and Engineering, Sun Yat-sen University, No. 132, Outer Ring East Road, Guangzhou, Guangdong Province 510006, China

Or Prof. Bo Wu (wubo28@mail.sysu.edu.cn)

School of Environmental Science and Engineering, Sun Yat-sen University, No. 132, Outer Ring East Road, Guangzhou, Guangdong Province 510006, China

**Running title:** PLA microplastics rewire soil food webs

**Supplementary Materials**

1. Experimental design

This study is built upon a core interaction interface encompassing “soil amoeba-bacteria-PLA microplastics” to systematically elucidate the ecological impact of PLA on the soil microbial web. Figure S1 outlines the multi-scale experimental framework. The experimental logic progresses through three interconnected phases.

(1) Field incubation: To assess PLA MP effects under environmentally relevant conditions, we established field plots with PLA MPs embedded in soil. This scale provides data on microbial community restructuring, metabolic functional shifts, and the formation of the PLA-associated “plastisphere”. Based on these observations, a key responder, the soil amoeba *Dictyostelium discoideum*, was identified and selected as the model organism for subsequent mechanistic investigation.

(2) Exposure assays: Laboratory soil microcosm and agar plate experiments were conducted under controlled conditions to precisely quantify the fitness costs (spore production, morphology change) and behavioral responses (avoidance) of amoebae to PLA, while preserving the biological complexity of the soil matrix.

(3) Dynamic behavioral dissection: This scale included assessing the degradation capacity of amoebae towards PLA, measuring alterations in their chemotaxis and direct predation efficiency, and dissecting the indirect interactions within the amoeba-bacteria-PLA MP interface. The arrows in Figure S1 illustrate the tiered and progressive logic of this research framework.

In summary, we screened for perturbed amoebae and their prey from field observations (Phase 1), tested these findings under controlled complexity (Phase 2), and further deconstructed them into fundamental mechanisms (Phase 3).

**2. Supplementary Methods**

To further evaluate the effects of 3 mm PLA particles on fruiting body formation and distribution, additional experiments were conducted based on the previous findings. The effects of six polymers on fruiting body formation of amoeba *D. discoideum* QS9 were compared in both SM/5 agar and soil microcosms. All non-biodegradable polymers were used as 3 mm particles. Specifically, the polylactic acid (PLA) was sourced from NatureWorks LLC (USA), the polyamide (PA) and polyethylene terephthalate (PET) were sourced from DuPont (USA), the polyvinyl chloride (PVC) was sourced from Tianjin Dagu Chemical Co., Ltd. (China), and the polypropylene (PP) and polyethylene (PE) were sourced from Sinopec Group (China).

Fruiting bodies were imaged using a Canon camera (EOS 800D), with particular attention paid to whether amoebae exhibited avoidance behavior toward the 3 mm microplastics. On day 7, sorus of fruiting bodies in the soil microcosms were gently collected using sterile inoculation loops, and the harvested spores were quantified using KK2 buffer.

**Table S1.** Soil parameters measured in this study, presented as mean ± standard deviation.

| **Soil parameters** | **Measured value (n=3)** |
| --- | --- |
| Moisture (%) | 15.5 ± 0.1 |
| pH | 7.0 ± 0.2 |
| TC (g kg^-1^) | 15.6 ± 0.4 |
| TN (g kg^-1^) | 1.24 ± 0.04 |
| TS (g kg^-1^) | 0.20 ± 0.01 |
| TH (g kg^-1^) | 6.6 ± 0.3 |
| C/N | 12.67 ± 0.65 |
| C/H | 2.36 ± 0.04 |
| Al (mg kg^-1^) | 6494 ± 130 |
| Cd (mg kg^-1^) | 6.67 ± 0.18 |
| Si (mg kg^-1^) | 2199 ± 58 |
| Fe (mg kg^-1^) | 17349 ± 190 |
| Cu (mg kg^-1^) | 15.21 ± 0.34 |
| As (mg kg^-1^) | 43.74 ± 0.81 |
| < 0.002 mm (%) | 4.7 ± 0.2 |
| 0.02 - 0.002 mm (%) | 13.5 ± 0.1 |
| 0.05 - 0.02mm(%) | 12.9 ± 0.3 |
| > 0.05 mm (%) | 68.9 ± 0.2 |

**Table S2.** Soil bacterial strains and isolation sources used in this study.

| **Bacterial strain** | **Isolation source** |
| --- | --- |
| *Klebsiella pneumoniae* KpGe | Soil |
| *Paracoccus denitrificans* DSM413 | Soil |
| *Lysinibacillus varians* GY32 | Soil |
| *Brevibacillus laterosporus* AMC797 | Soil |
| *Sphingobium hydrophobicum* C1 | Soil |
| *Xanthomonas campestris* ATCC33913 | Soil |
| *Pseudomonas fluorescens* Pf-5 | Soil |
| *Paenibacillus polymyxa* ATCC842 | Soil |
| *Shewanella decoloratiris* S12 | Activated sludge |

**Table S3.** Molecular weight parameters of pristine and amoebae-co-cultured PLA MPs (3 mm).

| **Sample** | ***M*_n_ (g/mol)** | ***M*_w_ (g/mol)** | ***M*_z_ (g/mol)** |
| --- | --- | --- | --- |
| PLA microplastics | 66663 | 99093 | 145591 |
| PLA MPs co-cultured with amoebae | 52246 | 87006 | 132094 |

**Figure S1.** Schematic flow chart of experimental design.

**Figure S2.** Daily maximum and minimum temperature variations during the field incubation experiment. Refer to Section 2.1 in Methods.

**Figure S3.** Relative abundance of soil microbial communities across different domains under PLA MPs treatment.

**Figure S4.** Alpha diversity (richness) across biological domains in response to PLA MP treatments.

**Figure S5.** Alpha diversity (Chao1) across biological domains in response to PLA MP treatments.

**Figure S6.** Compositional changes of soil protists under PLA MPs insertion revealed by heatmap. (A) Phylum level. (B) Class level. (C) Order level. (D) Family level.

**Figure S7.** Recorded growth morphology of amoebae under PLA MP exposure. (A) On SM/5 agar plates. (B) In a soil microcosm.

**Figure S8**. Gel permeation chromatography (GPC) molecular weight distributions of PLA microplastics in soil. (a) GPC chromatogram of PLA microplastics in the control group; (b) GPC chromatogram of PLA microplastics in the treatment group. The molecular weight parameters of PLA microplastics, including number-average molecular weight (*M*_n_), weight-average molecular weight (*M*_w_), and Z-average molecular weight (*M*_z_), were determined by GPC analysis.

**Figure S9.** Schematic of droplet mixing and labeling for the supplementary chemotaxis assay. The diagram illustrates the experimental groups, indicating the presence or absence of amoebae, food bacteria, and PLA MPs at specified concentrations. This supplementary assay was performed on non-nutrient agar plates. Instead of counting amoebae during migration, it specifically recorded the fruiting body formation at the bacterial colonization site after chemotaxis completion, with the outcome quantified by the total spores.

**Figure S10.** Initial setup of the supplementary chemotaxis assay. (A) *D. discoideum* spores were pre-incubated with two particle sizes of PLA MPs (A0: control, no MPs. A1–A4: 100 μm PLA MPs at 50, 500, 1500, and 2500 mg/kg. A5–A8: 10 μm PLA MPs at the same concentrations). (B) *K. pneumoniae* KpGe was pre-incubated with two particle sizes of PLA MPs (B0: control, no MPs. B1–B4: 100 μm PLA MPs. B5–B8: 10 μm PLA MPs. all at concentrations of 50, 500, 1500, and 2500 mg/kg).

**Figure S11.** Supplementary chemotaxis assay demonstrates the inhibitory effect of PLA microplastics on amoeba chemotaxis and predation. (A) Schematic diagram of the experimental procedure. A0-A8 and B0-B8 are defined as in Figure S8. (B) Quantification of total spores in fruiting bodies. The results show that pre-mixing PLA MPs either with amoebae or with bacteria significantly impaired the predatory efficiency of amoebae and reduced the final total spores.

**Figure S12.** Morphology of amoebae grown on non-nutrient agar under PLA MP exposure.

**Figure S13.** Fluorescence labeling of *Dictyostelium discoideum* under normal predation conditions. Trophozoites of *D. discoideum* were stained and mixed with amoebae and the food bacteria. From left to right: Bright field: Co-culture plate of amoeba and food bacteria (scale bar = 890 μm). Amoebae distribution: Uniform distribution of Calcofluor White-stained trophozoites across the agar plate (amoebae only). Bacterial fluorescence: GFP-labeled *K. pneumoniae* KpGe on the agar plate. Merge (Blue + Green): Combined fluorescence channels.

**Figure S14.** System dynamics in the presence of single or multiple 3 mm PLA microplastics. The panel documents the tripartite interactions between amoebae, bacteria, and PLA MPs, recorded with a Canon EOS 800D.

**Figure S15.** Distinct morphogenetic response of *D. discoideum* to PLA MPs. The fruiting bodies of *D. discoideum* bypassed the 3 mm PLA MP and formed an inhibition zone specific to protist-bacteria interactions.

**Figure S16.** Distinct morphogenetic response of *D. discoideum* to glass beads. The 3 mm glass beads served as the control, representing the objective state of spherical objects contacting the agar surface. In contrast to their interaction with the beads, the fruiting bodies of *D. discoideum* did not bypass the PLA MPs but grew immediately adjacent to them during development, including the aggregation stage.

**Figure S17.** Comparative analysis of total amoeba spores under different 3 mm microplastics. In a soil microcosm experiment, 3 mm particles of PLA, PET, PA, PP, PE, and PVC did not exert significant effects on the total spore production of amoebae.

**Figure S18.** Differential inhibitory effects of PLA MPs and lactic acid on bacteria.

(A) The effect of spherical objects on *K. pneumoniae* on the plate showed no inhibitory effect. The effect of 3mm PLA MPs on *K. pneumoniae* on the plate demonstrated significant inhibition. The effect of standard lactic acid on *K. pneumoniae* on the plate exhibited significant inhibition. (B) The gradient inhibitory effect of standard lactic acid solutions (pH=2–6) dropped at the center of the plate on *K. pneumoniae*.

**Figure S19.** Behavior of the food bacteria *Klebsiella pneumoniae* KpGe after co-incubation with two sizes of PLA MPs. (A) 100 μm PLA MPs. (B) 10 μm PLA MPs. Imaging reveals that both sizes of PLA MPs disrupted the bacterial network across the spatial domain.
